# Digital Phenotyping for Real-Time Monitoring of Nonsuicidal Self-Injury: Protocol for a Prospective Observational Study

**DOI:** 10.2196/53597

**Published:** 2024-02-08

**Authors:** Chan-Young Ahn, Jong-Sun Lee

**Affiliations:** 1 Department of Psychology Kangwon National University Chuncheon-si Republic of Korea

**Keywords:** nonsuicidal self-injury, NSSI, digital phenotyping, digital phenotype, wearable device, wearable, wearables, wrist worn, mood, emotion, emotions, heart rate, step, sleep, machine learning, multilevel modeling, ecological momentary assessment, EMA, self-injury, self-harm, psychiatry, psychiatric, mental health, predict, prediction, predictions, predictor, predictors, predictive

## Abstract

**Background:**

Nonsuicidal self-injury (NSSI) is a major global health concern. The limitations of traditional clinical and laboratory-based methodologies are recognized, and there is a pressing need to use novel approaches for the early detection and prevention of NSSI. Unfortunately, there is still a lack of basic knowledge of a descriptive nature on NSSI, including when, how, and why self-injury occurs in everyday life. Digital phenotyping offers the potential to predict and prevent NSSI by assessing objective and ecological measurements at multiple points in time.

**Objective:**

This study aims to identify real-time predictors and explain an individual’s dynamic course of NSSI.

**Methods:**

This study will use a hybrid approach, combining elements of prospective observational research with non–face-to-face study methods. This study aims to recruit a cohort of 150 adults aged 20 to 29 years who have self-reported engaging in NSSI on 5 or more days within the past year. Participants will be enrolled in a longitudinal study conducted at 3-month intervals, spanning 3 long-term follow-up phases. The ecological momentary assessment (EMA) technique will be used via a smartphone app. Participants will be prompted to complete a self-injury and suicidality questionnaire and a mood appraisal questionnaire 3 times a day for a duration of 14 days. A wrist-worn wearable device will be used to collect heart rate, step count, and sleep patterns from participants. Dynamic structural equation modeling and machine learning approaches will be used.

**Results:**

Participant recruitment and data collection started in October 2023. Data collection and analysis are expected to be completed by December 2024. The results will be published in a peer-reviewed journal and presented at scientific conferences.

**Conclusions:**

The insights gained from this study will not only shed light on the underlying mechanisms of NSSI but also pave the way for the development of tailored and culturally sensitive treatment options that can effectively address this major mental health concern.

**International Registered Report Identifier (IRRID):**

DERR1-10.2196/53597

## Introduction

Nonsuicidal self-injury (NSSI), defined as deliberate self-inflicted bodily harm without suicidal intent [1], is a major global mental health concern. A recent epidemiological study found that the estimated lifetime prevalence of NSSI in adults was 4.86%, with younger age being more associated with NSSI [2]. According to a meta-analysis, the pooled prevalence of NSSI in young adults was 13.4% [3], and epidemiological studies indicate that 1 in 5 young adults engage in NSSI at least once before the age of 25 years [4,5].

Research has shown that NSSI is associated with academic and interpersonal difficulties [6,7], substance dependence [8], rehospitalization [9], and an increased risk for psychiatric disorders [10-14]. Remarkably, NSSI, despite its absence of initial suicidal intent, is a strong predictor of future suicidal thoughts and behaviors, independent of psychiatric disorders [15-18].

A recent scoping review [19] found that most mobile apps designed to intervene in NSSI were effective in reducing the frequency of urges to self-harm. Similarly, a systematic review found preliminary evidence that psychological interventions for self-harm are associated with reductions in self-injurious thoughts and behaviors, with positive treatment effects also found for suicidal ideation [20]. In addition, psychotherapeutic interventions for NSSI have shown efficacy in improving global functioning and reducing NSSI and depressive symptoms [21], with significant improvements in hopelessness and problem-solving [22]. Given the efficacy and effectiveness of interventions for NSSI, it is important to detect people with self-injury behavior early and provide NSSI-specific interventions.

Despite the pressing need for early detection and intervention for NSSI, it is a worrying reality that a majority of people with self-injury behavior do not seek medical care or help, including psychotherapy [2,23-25]. Furthermore, individuals engaging in self-injurious behaviors often face issues of stigma and social undesirability, making it essential to explore non–face-to-face diagnostic and intervention approaches [26-28]. Additionally, retrospective self-reported methods to assess self-injury may be limited by recall bias [29,30], failing to capture the transient nature of self-injurious thoughts, especially in adults, where the frequency and intensity of such thoughts can fluctuate even within a single day [31]. Recognizing that traditional clinical- or laboratory-based methodologies have limitations, there is a pressing need to detect and prevent NSSI at an early stage.

There is an urgent need for rigorous descriptive research, especially observational research to measure change over time and document various aspects of NSSI behavior [32]. Understanding NSSI requires a systematic and meticulous approach to longitudinal and real-time data collection to enable researchers to produce information of a descriptive nature on the occurrence of NSSI. Designating information on NSSI behavior as being of a “descriptive nature” refers to the detail and comprehensiveness of our understanding; for NSSI, this encompasses factors such as its frequency, duration, triggers, and consequences. However, there is still a lack of rigor in understanding when, how, and why self-injury occurs in everyday life [33]. This gap in knowledge largely persists because most NSSI research has relied on cross-sectional designs, which may not capture the dynamic nature of NSSI behavior over time. While there are some longitudinal studies, they often use long observation windows to identify developmental risk factors, providing insights into who is at higher risk of self-injury relative to others over extended periods (ie, at a between-person level) [34]. These methods focus on long-term trends rather than immediate occurrences of NSSI. In other words, these methods may not have the temporal precision to detect imminent risk of self-injury in daily life, occurring within minutes or hours.

Digital phenotyping, as an approach, offers the potential to predict and prevent NSSI in everyday life by assessing an objective and ecological source of measurements at multiple points [35,36]. By leveraging this technology, researchers can collect real-time data on an individual’s self-injury–related markers, such as mood, sleep condition, and physiological responses such as heart rate [37-39]. This comprehensive data set enables a deeper understanding of the complex factors contributing to NSSI behaviors, ultimately facilitating more accurate prediction, early detection, and intervention strategies.

Digital phenotyping techniques, using smartphone and wearable devices, encompass 2 types of data: active and passive. Active data, such as data obtained with ecological momentary assessment (EMA), involve self-reported questionnaires that requires conscious effort from users. Passive data, including heart rate and sleep condition, on the other hand, are collected automatically without any user input. Predicting a high risk of NSSI in daily life can prompt the delivery of early real-time interventions. Despite their potential, few studies have examined individuals’ dynamic NSSI patterns in daily life. This study aims to identify real-time predictors and explain an individual’s dynamic course of NSSI.

This study aims to use cutting-edge digital phenotyping techniques to identify young individuals at risk of NSSI and develop targeted interventions.

## Methods

### Study Design

This study will use a hybrid approach, combining elements of prospective observational research with non–face-to-face study methods. It will use a digital app to conduct continuous digital phenotype assessments of self-injury and suicide-related conditions, mood, physical activities (eg, walking), and sleep conditions. It will also use a wrist-worn wearable device (Apple Watch) to measure physiological state (eg, heart rate).

### Ethical Considerations

The protocol for this study has been approved by the institutional review board of Kangwon National University (KWNUIRB-2023-02-008-001) to ensure compliance with ethical guidelines and research protocols. All participants will be given an informed consent form and will review its contents before making a decision to take part. The consent form will contain information about the purpose of the study, procedures, potential risks of participation, compensation, and privacy guarantees. Each participant will have 3 EMA periods spaced 3 months apart. If the EMA compliance rate is 90% or higher, the reward will be KRW ₩70,000 (equivalent to approximately US $53), if it is 70% or higher, KRW ₩50,000 (approximately US $38), and if it is 50% or higher, KRW ₩30,000 (approximately US $23); the reward will be given in the form of a gift certificate. All collected data will be anonymized and analyzed to ensure individuals cannot be identified. The data will be exclusively used for research purposes and will not serve any other function. Subsequently, data files will be transferred to an external hard drive and securely stored within the principal investigator’s laboratory, secured under lock and key. Access to the data will be restricted to the principal investigator and authorized research staff, with the lab key held solely by the principal investigator.

### Participants

This study aims to recruit a cohort of 150 adults aged 20 to 29 years who have self-reported engaging in NSSI on 5 or more days within the past year. Participants to be recruited will own a smartphone and provide signed informed consent. Exclusion criteria for participants are as follows: presenting suicidal ideation and plans at a level requiring immediate intervention, that is, if the participant reports (1) suicidal thoughts with actual intent to attempt suicide or (2) has attempted suicide or taken preparatory actions in the past month; current manifestation of psychotic or manic symptoms or substance use disorders; and currently receiving any other psychotherapy or counseling.

### Procedure

**Figure 1:**
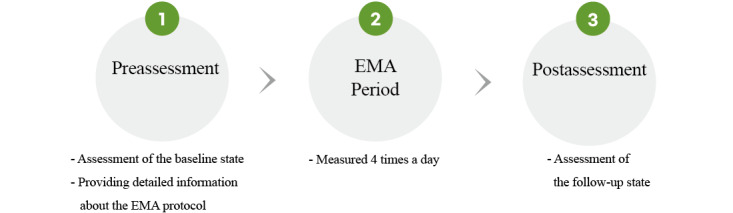
Study procedure. EMA: ecological momentary assessment.

Participants will be enrolled in a longitudinal study conducted at 3-month intervals spanning 3 long-term follow-up studies. The procedures shown in Textbox 1 and Figure 1 will be repeated for each study.

**Procedures for this longitudinal study conducted at 3-month intervals spanning 3 long-term follow-up studies.**

**Recruitment**

Potential participants meeting the criteria will be identified through targeted outreach efforts and community engagement. They will be provided with study information and invited to participate.

**Informed consent**

Participants who express interest in joining the study will be provided with detailed information regarding the study objectives, procedures, potential risks and benefits, and their rights as participants. An electronic version of consent in which participants click “yes” or “no” will be obtained from each participant prior to their inclusion in the study.

**Digital app enrollment**

After providing consent, participants will be guided through the process of enrolling in the app, named Dear My Mind, specifically developed for this study. This app will facilitate the collection of digital phenotyping data and serve as the primary mode of communication between the researchers and participants.

**Continuous digital phenotyping assessments**

1. Participants will complete a preassessment. The preassessment questionnaire will consist of the following items: the Functional Assessment of Self-Mutilation (FASM) [40] for an in-depth assessment of self-injurious behavior, Columbia Suicide Severity Rating Scale (C-SSRS) [41] to assess suicidality, Patient Health Questionnaire 9-item scale (PHQ-9) [42] to measure depression, Generalized Anxiety Disorder 7-item scale (GAD-7) [43] to measure anxiety, Korean version of the Pittsburgh Sleep Quality Index (PSQI-K) [44] to assess sleep conditions, Primary Care PTSD Screen for DSM-5 (PC-PTSD) [45] to assess posttraumatic stress disorder symptoms, WHO-5 Well-Being Index [46] to measure well-being, Brief Resilience Scale (BRS) [47] to measure levels of resilience, Self-Harm Inventory (SHI) [48] to measure deliberate self-harm, and Personality Assessment Inventory–Borderline Features Scale (PAI-BOR) [49] for the assessment of borderline personality disorder tendencies.
2. Active data will be obtained using ecological momentary assessment. Participants will provide active data regarding their mood, sleep, and self-injury behavior patterns 3 times each day. Additionally, they will be asked to record their mood daily at night time.
3. Passive data will be obtained using a wearable device. All participants will wear a wrist-worn wearable device, specifically an Apple Watch, for 14 days to passively collect data. This will include data related to activity levels, heart rate, and other relevant measures.

**Postassessment and debriefing**

The same questionnaire used for the preassessment will be administered for the postassessment. As compensation, participants with an overall compliance rate of 50% or more will receive KRW ₩30,000 (approximately US $23), participants with an overall compliance rate of 70% or more will receive KRW ₩50,000 (approximately US $38), and participants with an overall compliance rate of 90% or more will receive KRW ₩70,000 (equivalent to approximately US $53). Compensation will be based on 56 measurements collected over the 14 days.

### Measures

#### Active Assessment

**Figure 2:**
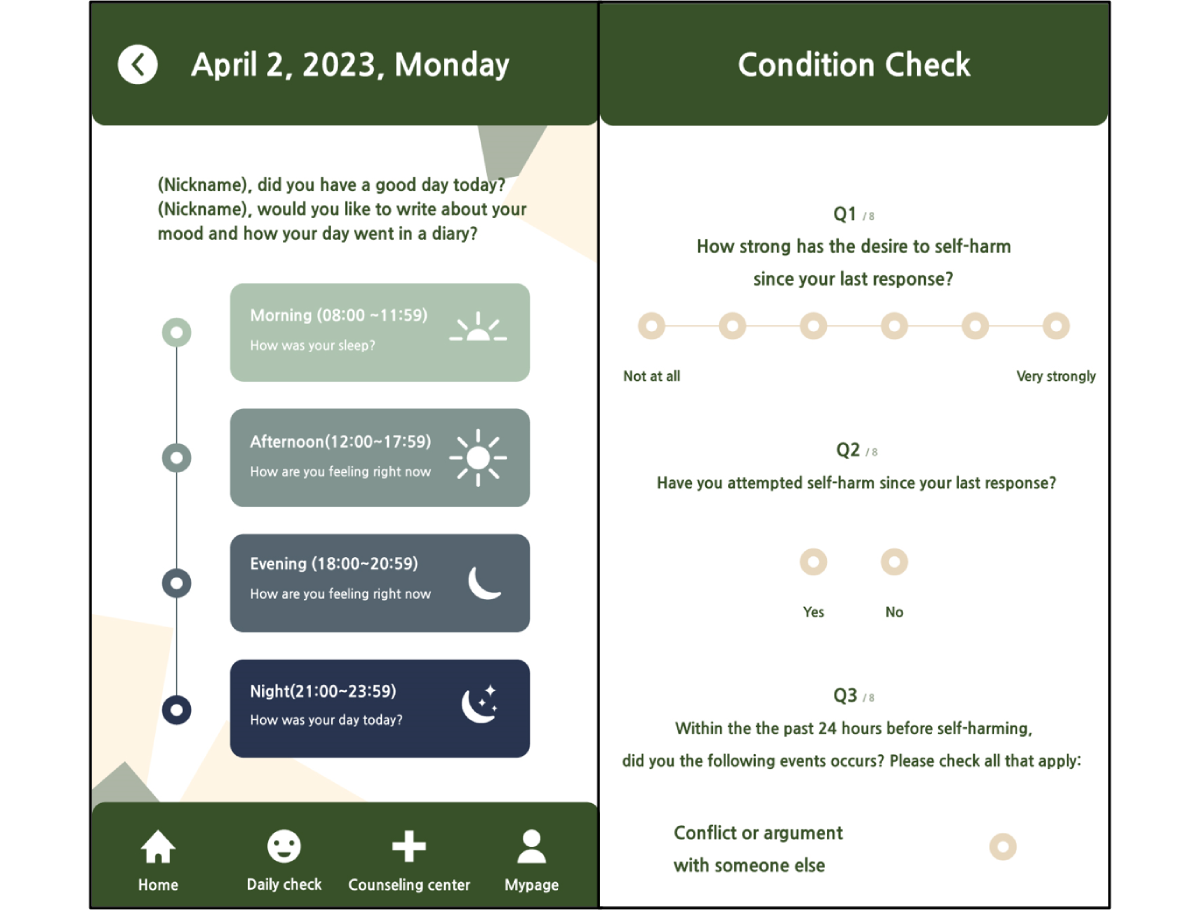
Dear My Mind app (under development).

EMA will be conducted via a smartphone app called Dear My Mind (currently under development; Figure 2). The EMA aims to capture real-time data on participants’ self-injury and suicidality, as well as mood appraisals. Participants will be prompted to complete the self-injury and suicidality questionnaire and the mood appraisal questionnaire 3 times a day for a duration of 14 days: at noon, in the evening, and at night (Table 1). Each morning, participants will be asked to answer a questionnaire regarding their sleep patterns from the previous night. This questionnaire will provide additional information about participants’ sleep conditions to complement the objective sleep measures collected. Furthermore, participants will be asked to complete a mood diary each night (the Mood Diary section below presents detailed information).

**Table 1:** Ecological momentary assessment schedule.

	Sleep condition check	Mood condition check	Self-injury and suicidality check	Mood diary
9 AM to 10 AM	✓			
Noon to 1 PM		✓	✓	
6 PM to 7 PM		✓	✓	
9 PM to midnight		✓	✓	✓

#### Self-Injury and Suicidality Questionnaire

The visual analog scale (VAS) is a rating of the participant’s perceived level of “self-injurious & suicidal thoughts and urges.” It is constructed on a horizontal line with “not at all” at one end and “very much” at the other (on a 7-point Likert scale). It can quantify a participant’s self-injurious and suicidal thoughts and urges and is useful for capturing changes within individuals rather than differences between individuals. In addition, if the participant has attempted self-injury since the last response, a text box prompts the participant to indicate the method of the attempt. There are also additional questions about antecedent events, antecedent emotions that occurred within the 24 hours prior to the self-injury, and experiences immediately following the self-injury. If the participant indicates that they have attempted suicide since their last response, they will be classified as high risk and the monitoring researcher will contact them immediately to refer them to a local mental health clinic or counseling center for immediate help.

#### Mood Appraisal Questionnaire

Participants will be instructed to respond to the VAS 3 times a day about the emotions they are experiencing at that moment. The mood VAS will be constructed on a horizontal line with “not at all” at one end and “very much” at the other (on a 9-point Likert scale). Specifically, participants will be asked about their feelings of depression, sadness, anxiety, fear, loneliness, rejection/hurt, anger at self, anger at others, shame, and emptiness.

#### Mood Diary

Participants will be asked to engage in daily mood diary entries using a free-text box. Each night, participants will be prompted to write about an event they experienced during the day, how it affected their mood, and the strategies they used to resolve it. Participants receive clear instructions regarding the purpose and content of the mood diary entries. A free-text format provides participants the flexibility to express their thoughts and feelings in their own words, allowing for a more nuanced understanding of their mood dynamics. Additionally, we emphasize the importance of consistent and honest reporting to maximize the validity and reliability of the data collected. The purpose of the mood diary is to capture participants’ subjective experiences and emotions, as well as their efforts to manage and overcome daily challenges. Participants will be encouraged to be as detailed and specific as possible when describing the event, their emotional response, and their coping strategies. This information will provide valuable insights into the contextual factors that influence participants’ mood fluctuations and their adaptive responses. The daily nature of the mood diary entries allows for the examination of day-to-day variations in mood and the exploration of the relationship between specific events and emotional well-being. It provides a comprehensive picture of participants’ subjective experiences and contributes to the overall understanding of their digital phenotypes.

#### Sleep Condition Questionnaire

To complement the objective sleep data obtained from the wearable device, participants will also be administered the sleep condition questionnaire to gather subjective sleep measures. This questionnaire includes items related to bedtime and wake time, actual sleep duration, and subjective sleep quality. Participants will be asked to respond to the sleep condition questionnaire once a day, specifically each morning, providing information about their sleep experience during the previous night. By capturing participants’ subjective perceptions of their sleep, this questionnaire will help elucidate participants’ personal experiences and perceptions of their sleep quality and add a valuable subjective component to the objective sleep data collected. Participants will be encouraged to provide accurate and detailed responses to provide a comprehensive understanding of their sleep patterns and experiences. By combining objective sleep data from the wearable device with participants’ subjective reports, this study will gain a more comprehensive and holistic understanding of participants’ sleep conditions.

### Passive Assessment

The wearable device will be used to collect several types of passive data from participants. Previous studies have shown the psychometric validity of the wearable device in reliably collecting physiological data [50,51]. The types of passive data collected in this study are heart rate, heart rate variability, step count, and sleep patterns. More specifically, the wearable device’s photoplethysmography sensor will collect data on an individual’s heart rate and heart rate variability [51]. In addition, a combination of sensors, such as the accelerometer, gyroscope, altimeter, and GPS, will be used to count steps. Through a combination of the above sensors, we will also measure sleep patterns such as core sleep, deep sleep, and rapid eye movement (REM) sleep.

**Figure 3:**
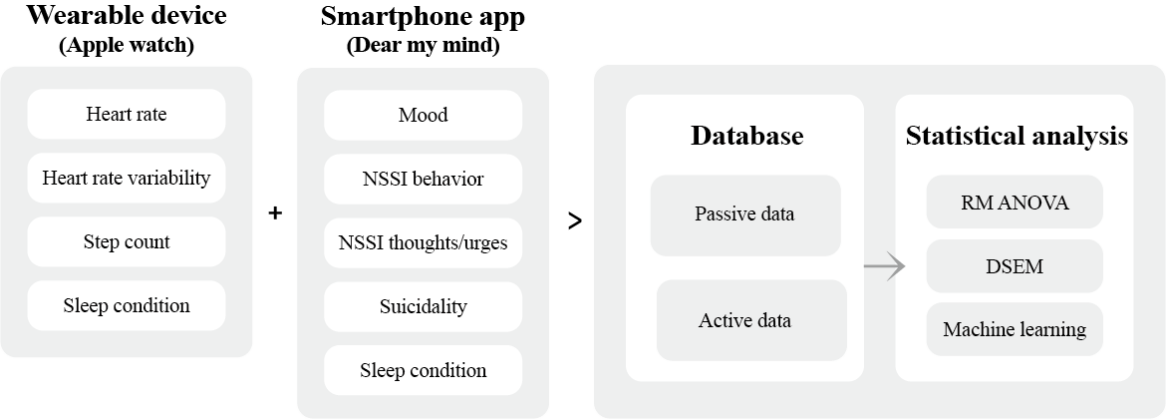
Flow of digital phenotyping. DSEM: dynamic structural equation modeling; NSSI: nonsuicidal self-injury; RM: repeated measures.

To ensure the protection of individual rights and privacy, passive data will only be accessed and used in accordance with Apple’s officially authorized data access protocols. Each research participant will be assigned a unique identifier, and their information will be uploaded to the database in a deidentified format. This process safeguards the confidentially of participants’ personal information. For the purpose of data collection, participants will be instructed to wear the wearable device on their wrist throughout the day, except during activities such as showering or charging. By leveraging the capabilities of the wearable device, this study aims to capture objective and continuous measurements of participants’ physiological variables and activity. These passive data sources will provide valuable insights into participants’ daily behaviors and help enhance the understanding of their digital phenotypes. Figure 3 shows an overview of the flow of digital phenotyping.

### Analytic Plan

This study will use a variety of analytical methods based on the study hypotheses and the characteristics of the data. The analytic methods described below will be used.

#### Basic Data Analysis

Initially, basic data analysis techniques such as 2-tailed independent *t* tests, linear regression analysis, repeated measures ANOVA, or other appropriate methods will be used to analyze the data and identify any significant changes or associations.

#### Dynamic Structural Equation Modeling

To examine dynamic changes at the within-individual level, dynamic structural equation modeling (DSEM) will be used. This method allows for the investigation of time-varying relationships and the identification of individual-level patterns of change over time. Specifically, we will examine whether an individual’s affective state, physiological state (heart rate, heart rate variability), and sleep state predict NSSI thoughts and behaviors from one observation window to the next. To address these 2-level structured data and to examine the temporal relationships between predictor variables and NSSI thoughts and behavior, multilevel vector autoregressive models were constructed within the framework of DSEM. This approach will enable us to investigate the extent to which time-varying variables at *t*–1 (eg, depressive affect) predict NSSI thoughts and NSSI behavior at *t*.

#### Machine Learning

To comprehensively analyze participants’ digital phenotypes and predict NSSI thoughts and behaviors, sparse logistic regression will be used. Logistic regression predicts the probability of being categorized into 1 of 2 groups when the response variable is binary. This model has the useful property that the estimated coefficients are log odds ratios, but the results are difficult to interpret when the number of variables becomes large. To overcome this problem, sparse logistic regression using the least absolute shrinkage and selection operator (LASSO) can be considered. This model simultaneously conducts feature selection and estimation, allowing for interpretation with a few selected important predictors. To evaluate prediction performance, we will divide the data set into a 30% subset for model estimation and a 70% subset for model training. Additionally, we will perform 5-fold cross validation to avoid overfitting the model. In this process, the full data set will be divided into 5 subsets, with 4 subsets used for training and the remaining subset used as an independent validation set. To solve the problem of imbalance in the training data, the synthetic minority oversampling technique (SMOTE) will be used. SMOTE is an oversampling technique that takes samples from a small number of classes in the data and adds random values to generate new samples to add to the data. This algorithm helps to address the issue of the overfitting problem caused by random oversampling [52].

#### Other Measures

Measures including accuracy, precision, sensitivity, specificity, and area under the curve will be calculated to explore the prediction performance of sparse logistic regression.

The outcome variables to be entered into the model are NSSI thoughts and behaviors, which will be coded dichotomously (0: absent; 1: present). The predictor variables to be included in the model as interval averages are step count, heart rate, and heart rate variability. These variables will be binned according to the intervals at which the dependent variable, NSSI thoughts and behaviors, will be assessed. The mean value per bin will then be calculated and used as a predictor. For example, as active data collection occurs 4 times, at 9 AM, noon, 6 PM, and 9 PM, the numerous values in the passive data will be divided into 4 bins: 8:00 AM to 11:59 AM, noon to 5:59 PM, 6:00 PM to 8:59 PM, and 9:00 PM to 23:59 PM. For heart rate, values less than 30 or greater than 200 beats per minute (bpm) will be discarded. Heart rate will be calculated as the mean heart rate in bpm, and heart rate variability will be obtained via the SD of the normal-to-normal interval (SDNN), which is the SD of the NN interval and the square root of the variance. Subtracting the mean from each heart rate interval yields the SD of heart rate intervals. Regarding step count, the total step count, average, median, and SD for each period will be calculated. For sleep data, preprocessing will ensure only 1 value per day, representing 1 value per night. Sleep data preprocessing will include the average, median, and SD of core sleep, deep sleep, REM sleep, and total sleeping duration. The ratio of each sleep duration to the total sleep duration will also be calculated as a reference for sleep quality.

## Results

Participant recruitment and data collection started in October 2023. Data collection and analysis are expected to be completed by December 2024. The results will be published in a peer-reviewed journal and presented at scientific conferences.

## Discussion

The objectives of this study are to identify the factors that can forecast NSSI thoughts and behaviors in real time within an individual’s everyday experiences and to explore the dynamic course of NSSI. This study will be the first attempt in Korea to use digital phenotyping for predicting NSSI. In this project, we can quantify the digital phenotypes of NSSI in individuals by measuring daily mood, self-injury and suicidality, sleep state, and physiological data through the Dear My Mind app and a wearable device. By identifying specific digital phenotypes and analyzing within-individual variations over time, this study seeks to categorize NSSI groups that are relevant to Korean culture. Furthermore, the findings of this research are expected to improve the accuracy of NSSI assessment in psychotherapy. By incorporating digital phenotyping, clinicians will have access to objective and real-time data, enabling them to make more informed treatment decisions and personalized interventions based on individual needs. One approach to tailoring interventions is through the integration of digital phenotyping and intervention strategies [53]. For example, by leveraging multimodal digital signals, such as mobile sensing data, in combination with ground truth data like self-reported information using EMA, predictive models of NSSI risk can be developed. These models can be used to create personalized interventions that are specifically tailored to an individual’s unique risk factors and patterns of NSSI behavior. By integrating real-time data collection and analysis, these interventions can provide timely and contextually relevant support to individuals at risk for NSSI, potentially enhancing their effectiveness.

One of the major limitations of this study is that it targets individuals who have engaged in NSSI 5 or more days in the past year, yet the brief 14-day monitoring period may not comprehensively capture NSSI behaviors. This can lead to an imbalance in the training data [54] and should be addressed by expanding the observation period and using appropriate methodologies. Despite this limitation, this research will ultimately serve as a crucial foundation for advancing our knowledge of NSSI in the Korean context. The insights gained from this study will not only shed light on the underlying mechanisms of NSSI but also pave the way for the development of tailored and culturally sensitive treatment options that can effectively address this major mental health concern.

## Abbreviations

bpm: beats per minute
DSEM: dynamic structural equation modeling
EMA: LASSO: least absolute shrinkage and selection operator
NSSI: nonsuicidal self-injury
REM: rapid eye movement
SMOTE: synthetic minority oversampling technique
VAS: visual analog scale

## Data Availability

The data sets generated and analyzed during this study are not publicly available because this project is currently in progress, but they are available from the corresponding author upon reasonable request.
